# Identification of Proteins Associated with Polyhydroxybutyrate Granules from *Herbaspirillum seropedicae* SmR1 - Old Partners, New Players

**DOI:** 10.1371/journal.pone.0075066

**Published:** 2013-09-25

**Authors:** Evandro F. Tirapelle, Marcelo Müller-Santos, Michelle Z. Tadra-Sfeir, Marco A. S. Kadowaki, Maria B. R. Steffens, Rose A. Monteiro, Emanuel M. Souza, Fabio O. Pedrosa, Leda S. Chubatsu

**Affiliations:** Departamento de Bioquímica e Biologia Molecular, Universidade Federal do Paraná, Curitiba, Brazil; INRA Clermont-Ferrand Research Center, France

## Abstract

*Herbaspirillum seropedicae* is a diazotrophic ß-Proteobacterium found associated with important agricultural crops. This bacterium produces polyhydroxybutyrate (PHB), an aliphatic polyester, as a carbon storage and/or source of reducing equivalents. The PHB polymer is stored as intracellular insoluble granules coated mainly with proteins, some of which are directly involved in PHB synthesis, degradation and granule biogenesis. In this work, we have extracted the PHB granules from *H. seropedicae* and identified their associated-proteins by mass spectrometry. This analysis allowed us to identify the main phasin (PhaP1) coating the PHB granule as well as the PHB synthase (PhbC1) responsible for its synthesis. A *phbC1* mutant is impaired in PHB synthesis, confirming its role in *H. seropedicae.* On the other hand, a *phaP1* mutant produces PHB granules but coated mainly with the secondary phasin (PhaP2). Furthermore, some novel proteins not previously described to be involved with PHB metabolism were also identified, bringing new possibilities to PHB function in *H. seropedicae*.

## Introduction

Polyhydroxybutyrate (PHB) is an aliphatic polyester biosynthesized by several bacteria as a means of carbon storage and source of reducing equivalents [1]. PHB is usually produced under conditions of carbon oversupply and low levels of other nutrients including nitrogen, phosphate and oxygen [2]. A correlation between PHB production and improvement in bacterial survival under stress conditions or in competitive environments has been reported [3], [4]. This polymer synthesis is dependent on, at least, three enzymes: 3-ketothiolase, acetoacetyl-CoA reductase and PHB synthase, encoded by *phbA*, *phbB* and *phbC* genes, respectively [5]. When carbon/energy is necessary, the polymer is degraded by a PHB depolymerase enzyme, encoded by *phaZ* [5].

The PHB polymer is stored as intracellular insoluble granules coated with proteins reaching about 0.5 to 2% of the granule weight [1], [6]. Previous reports have also indicated the presence of lipids coating PHB granules (reviewed by [6]), however a recent report suggested otherwise [7]. Four types of proteins have been described to be associated with the PHB granules: phasins, PHB synthase, PHB depolymerase, and the transcriptional regulatory protein PhaR/PhbF [8]. Phasins are the main protein coating the PHB granule, preventing its coalescence. On the other hand, association of the transcriptional repressor PhaR/PhbF protein with the granule may suggest a mechanism where PhaR/PhbF is displaced from the promoter region of target genes by binding to nascent PHB granule thus allowing transcription of genes involved in its metabolism [8]. In addition to these four, other proteins have been found associated with the PHB granule (reviewed by [1]), however their physiological role is not clear.

*Herbaspirillum seropedicae* is a diazotrophic β-Proteobacterium, which associates with graminae [9] and produces PHB [10], [11]. In *H. seropedicae*, thirteen genes potentially involved in PHB metabolism were identified [11], [12], including two *phbC, phaZ* and *phaP* genes with codes for synthases, depolymerases and phasins, respectively, and the *phbF* gene which encodes the regulatory protein [11]. It has been suggested that PHB has an important role in nitrogen fixation and in plant-bacteria interaction [3], [13], however the importance of PHB production for *H. seropedicae* was not assessed.

In this report we used a proteome approach to identify proteins associated with *H. seropedicae* PHB granules and determined the genes products involved in PHB metabolism. Two mutants were constructed (*ΔphbC1* and *ΔphaP1*) and analyzed for the role of their products on the PHB metabolism.

## Materials and Methods

### Reagents

All chemicals were Analytical or Molecular Biology grade and were purchased from Merck (Germany), Sigma (USA), J.T.Baker (Netherlands) or Invitrogen (USA). Restriction enzymes were from Fermentas (Lithuania) or Invitrogen (USA). Oligonucleotides were from IDT (USA). Reagents for RNAseq were from Life Technology (USA).

### Bacterial Strains and Growth Conditions

*Escherichia coli* strains TOP10 (Invitrogen, USA) and S17.1 [14] were used for cloning and conjugation procedures. They were grown at 37°C in LB, Terrific Broth, SOC or SOB medium [15]. *Herbaspirillum seropedicae* wild type SmR1 [16] and mutants strains were grown in NFbHP medium with 37 mM malate and 20 mM NH_4_Cl at 30°C [17].

### Identification of PHB Granule-associated Proteins

*H. seropedicae* strain SmR1 was cultivated in 500 mL of NFbHP medium with 37 mM malate and 20 mM NH_4_Cl at 30°C. At O.D._600_ of 1.0, cells were harvested by centrifugation (6,000×*g*, 10 min, 4°C). The cell pellet was washed once with potassium phosphate buffer (100 mM, pH 7.0), resuspended and sonicated in the same buffer. The PHB granules within the insoluble fraction were then purified by ultracentrifugation in two glycerol gradients as previously described [18]. The proteins associated with the PHB granules were removed by boiling the granule suspension with 1× Laemmli loading buffer for 5 min and analyzed using SDS-PAGE in Tris-glycine buffer [19]. Proteins were stained with Coomassie blue R-250 followed by a densitometric analysis for relative quantification. Three biological replicates were analyzed. The proteins were identified by peptide mass fingerprinting (PMF) in a MALDI ToF/ToF Autoflex II spectrometer (Bruker Daltonics, USA) as previously described [20], [21] using positive reflector mode, voltage acceleration of 20 kV, intervals of ion extraction of 150 ns and acquisition of 800–3200 m/z. Spectra analyses were performed using the FlexAnalysis 3.0 (Bruker Daltonics). Data on peptide fragmentation (MS/MS) was obtained using the LIFT method of FlexControl 3.0 (Bruker Daltonics). Database search was carried out using Mascot 2.3 against a local database of *H. seropedicae* predicted proteins. Error tolerance for PMF was 100 ppm and 0.3 Da.

### PHB Quantification

The amount of PHB was determined by methanolysis and GC-FID analysis as described previously [22]. Methanolysis was carried out with 5–10 mg of lyophilized bacteria in 2 ml of chloroform and 2 ml of methanol containing 15% sulphuric acid in borosilicate glass tubes with screw caps. As an internal standard, it was added 0.5 mg of benzoic acid per reaction. The reactions were incubated at 100°C for 3.5 h in a dry heating block. After cooling, 1 ml of distilled water was added and the tubes were mixed by vortex during 60 s. After phase partition, the upper aqueous phase was removed and the lower organic phase containing the resulting 3-hydroxybutyric methyl ester (Me-3-HB) was dried with Na_2_SO_4_ and analyzed by GC in a 450 GC Varian chromatograph with a CP-Sil-5 CB column (10 m×0.53 mm ID). Argon was used as carrier gas at a flow rate of 0.9 ml min^−1^. The injector was set at 250°C and the detector at 275°C. The oven temperature programmed was: initial temperature 50°C for 2 min, then from 50°C up to 110°C at a rate of 20°C min^−1^ and finally up 250°C at a rate of 20°C min^−1^. The PHB amount in each sample was normalized by the weight of the lyophilized bacteria and expressed as % of PHB/dry cell weight (dcw).

### *H. seropedicae* Mutants Construction

The *H. seropedicae phbC1* mutant was constructed by deleting the *phbC1* gene (Hsero_2999; genome GenBank accession number CP002039.1). DNA fragments containing about 370 bp of the regions upstream and downstream of *phbC* were amplified using primers which introduced a KpnI restriction site on the 3′ end of the upstream fragment (phbCFwUp 5′-TCAGTCGACCAGCAGTTTTGTTAGTC-3′ and phbCRvUp 5′-TCGGTACCTGTTCATGCTCGGATCTG-3′) and on the 5′ end of the downstream fragment (phbCFwDw 5′-TAGGTACCATAAAAAAGAGGGCGGTG-3′ and phbCRvDw 5′-AAGGATCCGATGCATGGATATCGAAG-3′). These DNA fragments were initially cloned separately into pTZ57R/T (ThermoScientific, USA), and then both fragments were ligated to each other using the KpnI site. The resulting DNA fragment containing a deletion of the whole *phbC* gene was then cloned into pK18mobsacB plasmid [23]. The resulting plasmid was introduced into *H. seropedicae* SmR1 by conjugation with the *E. coli* S17.1 as donor. A *H. seropedicae* mutant strain able to grow in presence of sucrose was isolated and analyzed, by DNA amplification, to confirm the deletion of the *phbC1* gene. The resulting strain was named *ΔphbC1*.

The *H. seropedicae phaP1* mutant was construct by deleting the *phaP1* gene (Hsero_1639) using a similar procedure as above. Primers introduced SalI and KpnI restriction sites at the 5′ DNA fragment (about 450 bp) (phaPFwUp 5′-TTGGTCGACTGCGGTACTTG-3′ and phaPRvUp 5′-TGGGGTACCTGAAACATCCTT- 3′) and KpnI and BamHI restriction sites at the 3′fragment (about 330 bp) (phaPFwDw 5′-TCTGGTACCTTGTCTGGCG-3′ and phaPRvDw 5′-TCTGGATCCGCCGATGTTC-3′). These fragments were ligated using the KpnI site. After cloning procedure and conjugation, a *H. seropedicae* mutant strain was selected and analyzed by DNA amplification to confirm *phaP1* gene deletion. This strain was named *ΔphaP1*.

### Transcriptome RNAseq Assay

*H. seropedicae* SmR1(wild type) was grown in NFbHP medium with 37 mM malate and 20 mM NH_4_Cl at 30°C for 6 hours to reach O.D._600_ of 0.8. The total RNA was isolated using RiboPure™-Bacteria Kit (Ambion). Samples were treated with DNase I (Ambion), and RNA was quantified using a NanoDrop apparatus (ThermoScientific, USA) and analyzed by gel electrophoresis. The total RNA was partially depleted from rRNA using the MICROBExpress™ Bacterial mRNA Enrichment Kit (Ambion). The cDNA libraries were constructed with 0.5 µg ribosomal depleted RNA samples using the SOLiD™ Whole Transcriptome Analysis Kit (Life Technologies) and the SOLiD™ Transcriptome Multiplexing Kit (Life Technologies). The emulsifier PCR and SOLiD run parameters followed standard Life Technologies protocols. Libraries of each biological replicates were sequenced twice independently. Reads were mapped against *H. seropedicae* genome reference sequence (Genbank accession number CP002039.1). Data processing and statistical analyses were performed using the CLC Genomics Workbench 5.1 and the results were expressed in RPKM (Reads Per Kilobase of exon model per Million mapped reads) [24].

### Bioinformatics Analyses

Protein molecular weight and pI were determined by PROTPARAM-ExPAsy [25]. Similarity between two proteins were determined using the EMBOSS Needle-Alignment tool at EMBL-EBI (www.ebi.ac.uk). Protein families and domains were obtained using Pfam (http://pfam.sanger.ac.uk/). Secondary structure was predicted using PSIPRED v3.0 [26].

## Results and Discussion

**Figure 1:**
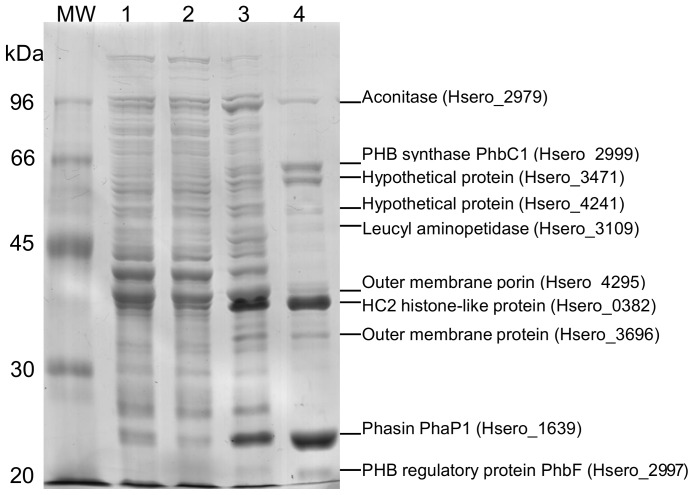
Electrophoretic pattern of PHB granule-associated proteins from *Herbaspirillum seropedicae* strain SmR1 in 10% SDS-PAGE. Lanes: MW – molecular weight markers, 1– crude protein extract, 2– soluble fraction, 3– insoluble fraction and 4– granule-associated proteins after granule purification. Proteins were Coomassie blue R-250 stained.

A proteome analysis was carried out on *H. seropedicae* in order to identify PHB granule-associated proteins using SDS-PAGE and mass spectrometry. The profile of the PHB granule-associated proteins is showed in Figure 1 (lane 4) and PMF-identified proteins are indicated in Table 1 (additional information is available as supplementary material, Fig. S1). Some of the identified proteins were already reported to be associated with PHB granules in other organisms [1]. These proteins included phasins, PHB synthase, PHB depolymerase, and the regulatory PhbF protein, all directly involved in PHB metabolism.

**Table 1:** Proteins associated with PHB granules of *H. seropedicae* identified by MALDI-TOF Mass Spectrometry.

Protein	M_r_ (kDa)^a^	Score^a^	Cov. (%)^a^	Matches^a^	Exp. M_r_ (kDa)^b^	%vol^c^	Gene Id^d^	Sequence^e^	Error (ppm)^a^
Aconitate hydratase (AcnA)	97.46	70	7	5/5	109	5	Hsero_2979	ADJ64467.1	40
PHB synthase (PhbC1)	64.70	63	9	4/4	69	12	Hsero_2999	ADJ64487.1	31
Non-conserved Hypothetical protein	61.47	62	11	5/8	64	14	Hsero_3471	ADJ64950.1	53
Conserved hypothetical protein	58.43	118	20	9/12	61	2	Hsero_4241	ADJ65710.1	66
Leucyl aminopeptidase	52.82	86	14	6/77	55	2	Hsero_3109	ADJ64593.1	67
PHB depolymerase (PhaZ1)	46.56	206	39	12/13	50	<1	Hsero_1622	ADJ63135.1	60
Outer membrane porin	39.37	120	33	8/11	42	5	Hsero_4295	ADJ65764.1	63
HC2 histone-like protein*	26.86	77	6	*	39	17	Hsero_0382	ADJ61907.1	24
Outer membrane porin (OmpA)	21.30	89	23	6/15	35	5	Hsero_3696	ADJ65174.1	52
Phasin (PhaP1)	19.79	163	79	11/26	24	34	Hsero_1639	ADJ63152.1	39
PHB regulatory protein (PhbF)	21.47	79	48	6/6	20	2	Hsero_2997	ADJ64485.1	8

a theoretical M_r_, expected scores, coverage, numbers of matched peptides and errors of mass determination were calculated by Mascot (Matrix Science),

b experimental M_r_ were calculated based on the relative migration of each protein compared to molecular weight markers,

c the densitometric relative volume (%) of each band was calculated assuming the sum of intensity of all detected bands as 100%,

d the gene identification is the same as deposited in the Genbank (accession number CP002039.1),

e GenBank accession sequence.

* protein identified only by MS/MS analysis.

The ability of the regulatory *H. seropedicae* PhbF protein to bind PHB granule has been shown previously using a recombinant expressed PhbF protein [11], and our results now confirm the ability of this transcriptional repressor protein in binding PHB granule *in vivo*. Since PhbF binds to the promoter region of *phb*-related genes and also to PHB granules, the regulation of *phb* genes expression most likely involves disruption of PhbF-DNA and formation of PhbF-granule complexes as previously proposed [8].

**Figure 2:**
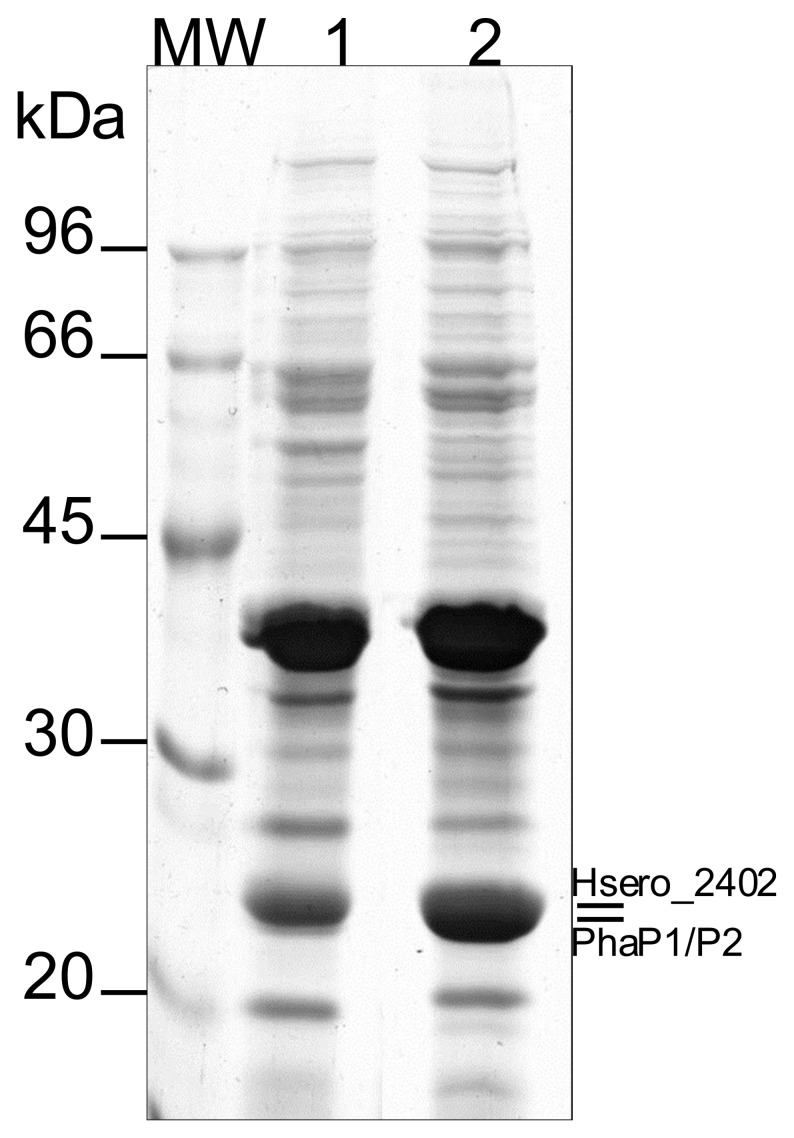
Electrophoretic pattern of PHB granule-associated proteins from *Herbaspirillum seropedicae* SmR1 and Δ*phaP1* strains in 10% SDS-PAGE. Lanes: MW – molecular weight markers; lane 1: granule-associated proteins after granule purification from the wild type strain SmR1; lane 2: granule-associated proteins after granule purification from the Δ*phaP1* mutant strain. Phasins are indicated on the right. PhaP1was identified in lane 1 whereas PhaP2 and Hsero_2402 were identified in lane 2. Proteins were Coomassie blue R-250 stained.

The most abundant protein associated with the *H. seropedicae* PHB granule is the phasin which has been described as responsible for preventing granule coalescence [27]. Genome analysis indicated two genes coding for phasins in *H. seropedicae* (Hsero_1639 and Hsero_4759) (GenBank accession number ADJ63152.1 and ADJ66220.1, respectively [12]). These genes code for 19.8 kDa proteins with pI of 6.7, sharing 58% identity and 74% similarity. Our analysis indicates that PhaP1 (encoded by Hsero_1639) is the main phasin coating PHB in *H. seropedicae*, since under our experimental conditions, PhaP2 (Hsero_4759) was not observed. However, absence of PhaP2 cannot be rule out since transcriptome analysis (RNA-seq) indicates that *phaP2* is expressed, although at a very low level when compared to *phaP1* (Table 2). A *phaP1* deleted mutant was constructed in order to verify if PhaP2 could substitute PhaP1 in coating PHB granules in *H. seropedicae*. The *ΔphaP1* mutant produces PHB indicating that synthesis of PHB is not impaired by *phaP1* deletion, however, the macroscopic aspect of the granule suspension is different compared to the wild type (data not shown), which may suggest a different morphology of the PHB granule. Protein analyses of the *ΔphaP1* PHB granule-associated proteins show a high concentration of PhaP2 indicating that in the absence of PhaP1, the second phasin PhaP2 is expressed and coats the PHB granules (Figure 2). Moreover, another protein associated with the PHB granule was also identified in the *ΔphaP1* mutant strain. Hsero_2402 (GenBank accession number ADJ63901.1) encodes a hypothetical protein with a calculated 21.6 kDa and pI of 6.2 and no conserved domain. It has 14.3% identity and 36% similarity with PhaP1 and 13.3% identity and 34.5% similarity with PhaP2. Although Hsero_2402 encoded protein shows low similarity with PhaP1 and PhaP2, prediction of secondary structure indicates a protein with high content of α-helix (supplementary material, Fig. S2) which may be involved in binding to PHB granule as reported [28]. Transcriptome analysis (RNA-seq) of the wild type strain (SmR1) indicated that both Hsero_2402 and PhaP2 (Hsero_4759) are expressed at the similar levels, and lower expression level as compared to that of PhaP1 (Hsero_1639) (Table 2). This difference in the transcription levels could favor the identification of PhaP1 and not the PhaP2 and Hsero_2402 by PMF in the wild type strain. Other organisms have been reported to carry more than one phasin, but their role on PHB metabolism differ. In *Ralstonia eutropha* five genes coding for phasins were identified and recently two more phasins were described [29]. Although all the seven genes are expressed during PHB synthesis, PhaP1 seems to be the main protein covering PHB granules [18], [29]. On the other hand, in *Sinorhizobium meliloti* two phasins are the main proteins associated with PHB granules [13]. The role of phasins in *H. seropedicae* and regulation of transcription of these genes are currently under investigation.

**Table 2:** Expression level of genes related to phasins in *Herbaspirillum seropedicae*.

Gene^a^	Expression value (RPKM)
Hsero_1639 (*phaP1*)	1184±294
Hsero_4759 (*phaP2*)	207±30
Hsero_2402	194±43

a the gene identification is the same as deposited in the Genbank (accession number CP002039.1).

The expression value corresponds to the average of RPKM of two biological replicates. RPKM is defined as RPKM = total gene reads per mapped reads (millions) × gene length (kb).

**Figure 3:**
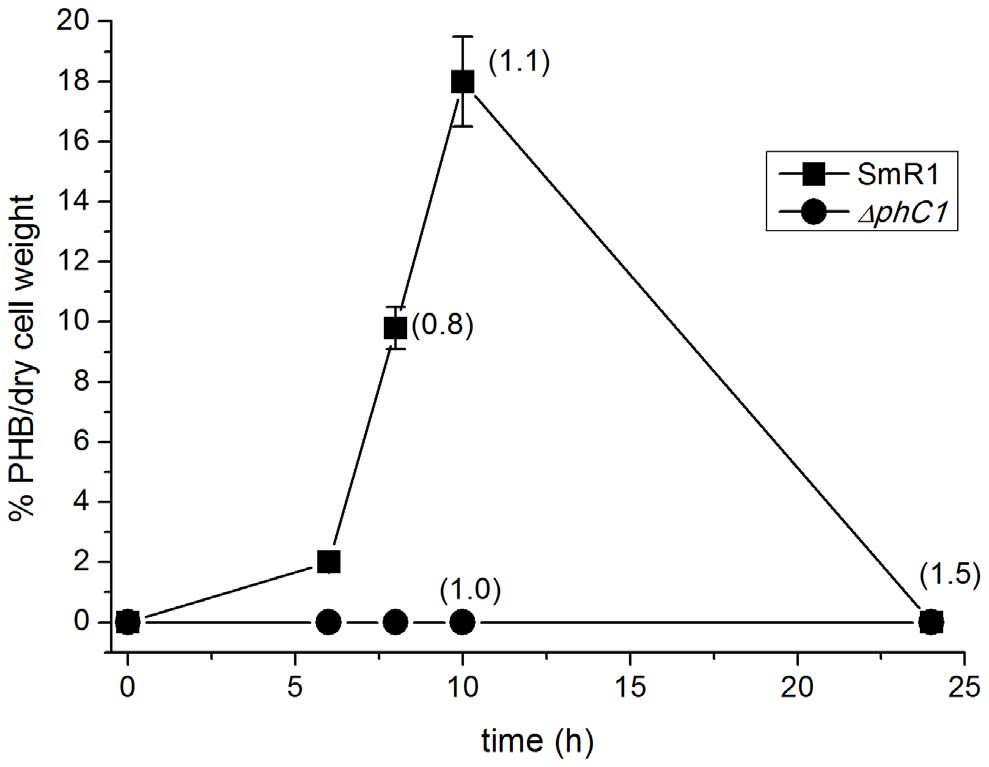
PHB production of *Herbaspirillum seropedicae* strains SmR1 (wild-type) and Δ*phbC1*. The quantity of PHB was determined as described in Material and Methods during different time of cell growth. Numbers in parenthesis indicate the OD_600_ of the cell culture at the indicated time of growth. Data represent the average of two biological replicates.

The third step on PHB biosynthesis relies on the PhbC synthase activity which has been found bound to PHB granule [1], [8]. *H. seropedicae* genome analysis indicated two genes coding for PhbC synthases: Hsero_2999, located upstream *phbB* and *phbF*, and Hsero_2405 located downstream a *dksA* homolog gene, whose putative product is a RNA polymerase-binding protein. These genes code for 64.7 and 64.9 kDa proteins, respectively, sharing 32% identity and 47% similarity. Although the location of Hsero_2999 close to *phbB* and *phbF* suggested it as the PHB synthase responsible for PHB synthesis in *H. seropedicae*, no other information was available. The Hsero_2999 coded protein represented about 12% of the PHB-associated proteins (Figure 1 and Table 1). In order to determine the role of Hsero_2999 on PHB metabolism, a *H. seropedicae* mutant (strain *ΔphbC1*) was constructed by gene deletion and PHB production was determined during cell growth (Figure 3). The *H. seropedicae* SmR1 (wild type strain) produced 18±2% of PHB/dry cell weight when reaching the stationary phase of growth. At late stationary phase, PHB was not detected in the wild type, most likely due to its degradation. On the other hand, PHB was not detectable in the *ΔphbC* mutant strain in any condition, reinforcing that Hsero_2999 indeed codes for the PhbC synthase responsible for PHB biosynthesis in *H. seropedicae.* This protein was called PhbC1.

Accumulated PHB can supply bacteria with carbon and energy when necessary. The PHB degradation relies on PHB depolymerase activity which are coded by *phaZ*. *H. seropedicae* has two *phaZ* genes: Hsero_0639 codes for a 45.5 kDa protein and Hsero_1622 which codes for a 46.6 kDa protein. These proteins share 45% identity and 62% similarity. The Hsero_1622 encoded protein was found bound to PHB granule (Table 1) therefore it is most likely the candidate for PHB degradation and was named PhaZ1. The observed amount of PhaZ1 bound to PHB was below 1% of total granule-associated proteins probably due to the growth stage of the culture which favors PHB production (Figure 3) rather than degradation.

In addition to PhbF, PhaP1, PhbC1 and PhaZ1, a leucyl aminopeptidase was also identified associated to PHB. This protein was found at low concentration in the *H. seropedicae* PHB granule (Table 1, Figure 1). Ren et al [30] also reported a similar protein associated with polyhydroxyalkanoate granules from *P. putida*. Although it has been suggested as an enzyme involved in turnover of granule-associated proteins, its role in PHB metabolism or granule biogenesis has not yet been demonstrated [30].

Our results also revealed novel proteins associated with the PHB granules in *H. seropedicae*. The second most abundant protein associated with PHB granules was a histone-like protein carrying a putative HC2 domain (Histone H1-like nucleoprotein HC2) encoded by Hsero_0382. The HC2 protein has been studied mainly in *Chlamydia* sp. It contains DNA- and RNA-binding activities, and has been shown to be involved in DNA condensation and regulation of both transcription and translation [31], [32]. The *Chlamydia* HC2 protein has several repeats of an amino acid pentamer containing arginine and lysine residues resulting in an uniform distribution of positive charges throughout the protein [31]. The HC2 homologue from *H. seropedicae* is a 282 amino acid protein with high content of alanine (45.4%), lysine (28%) and proline (11%), mainly in 15 pentameric repeats of AAAKK and AAPKK distributed throughout the protein and rendering positive charges evenly distributed. The high pI (11.8) of HC2 homologue leads to an unusual migration rate in SDS-PAGE (migrates as 40 kDa as compared to the calculated 26.9 kDa). Histone–like proteins associated with PHB granules have also been described for PhaF from *P. putida* and PhaM from *R. eutropha* [33], [34]. These proteins show alanine/lysine repeats located at the C-terminal portion of the protein, but in contrast with *H. seropedicae* HC2 homologue, different motifs at the N-terminal portion: a phasin domain in PhaF and potential transmembrane domains in PhaM. These proteins have been implicated with PHB granules association and their appropriated segregation during cell division [33], [34]. Although, the *H. seropedicae* HC2 homolog has not other known domains, a similar role in PHB granule segregation may not be rule out.

The hypothetical protein Hsero_3471 was also identified attached to the PHB granules at high levels (14% of the total granule-associated protein). Two genes encoding proteins homologous to Hsero_3471 were located in the *Cupriavidus necator* N-1 genome [35] (protein CNE_1c33190 - identity 48% and protein CNE_BB1p06610 - identity 45%). These proteins do not show any significant homology to known domains, with exception of a very low homology (e-value of 0.26) to the eukaryotic MACPF protein (membrane attack complex perforins). Analysis of the promoter region of Hsero_3471 indicates a potential PhbF-binding site [11] located at −190 position from the start codon, suggesting that its expression is regulated by PHB metabolism.

Two proteins related to porins have been also found associated with the *H. seropedicae* PHB granules: Hsero_4295 and Hsero_3696 which share similarity to OmpC and OmpA, respectively. Together these proteins represent approximately 10% of total PHB-associated proteins. Although they were not identified in a previously reported membrane proteome analysis of *H. seropedicae* SmR1 [21], a possible contamination during PHB granules extraction cannot be ruled out. Transcriptomic assays (RNAseq) indicated that both genes are heavily expressed (RPKM of 18591±5029 and 5756±220 for Hsero_4295 and Hsero_3696, respectively), corresponding to the highest expressed OmpC-like and OmpA-like coding genes in *H. seropedicae* under the assay conditions. Interaction of PHB of short chain (cPHB) with *E. coli* OmpA and its potential role on protein sorting and folding has been described [36] which could explain the binding of these *H. seropedicae* proteins to PHB granules upon extraction.

The conserved hypothetical protein Hsero_4241, a minor component of the PHB granule proteins, contains the domains CYTH (adenylyl cyclase family) and CHAD (conserved histidine α-helical domain) [37] with significant scores as determined by the Pfam analysis. The CHAD domain contains histidines that are predicted to either chelate metals or act as phosphoaceptors [37], and proteins containing domains CYTH-CHAD, as Hsero_4142, are thought to be organo-phosphate-recognizing enzymes that may have an active role in nucleotide and polyphosphate metabolism [37].

Interestingly, the aconitase AcnA was identified attached to PHB granules in *H. seropedicae*. To the best of our knowledge, it is the first time that the presence of an aconitase is reported on PHB granules. Aconitases can occur in two forms in bacteria. Under iron-replete condition they are catalytically active, containing a [4Fe-4S] cluster, but under iron-starvation or when the concentration of reactive oxygen species increases, the [4Fe-4S] cluster is lost and the apo-aconitase is formed [38]. Gupta and co-workers have shown that the apo-aconitase from *E. coli* has its surface hydrophobicity increased and that GroEL rescues the apo-protein from aggregation [39]. In this case, due the hydrophobicity of PHB, it is possible that the aconitase could bind to the granules non-specifically. Conversely, if one considers that the active aconitase is associated, it is expected that the sequestration by PHB may have metabolic consequences to the cell, mainly through modulation of metabolic flux of the TCA cycle.

The extraction of native granules by mild manipulation in glycerol gradients, associated with mass spectrometry analysis allowed identification of known and new proteins associated with PHB granules from *H. seropedicae*. Although contamination with proteins not related to PHB metabolism could not be ruled out, we decide to analyze those proteins found at higher concentration with the PHB granules, in order to minimize proteins unspecifically bound to PHB granule. Moreover the identified proteins were also compared to those previously described in the total proteome as well as the membrane proteins of *H. seropedicae* wild type strain [21], [40]. Except for PhaP1 (Hsero_1639) and PhbF (Hsero_2997), none of the proteins identified in this work has been described before using a proteome approach, suggesting a lower overall amount when compared to the whole protein sample or those found within the cellular membrane. These reinforces the specificity of the proteins described in this work related to PHB metabolism.

Among the different proteins identified are those which have been previously found to be involved in *H. seropedicae* PHB-metabolism such as PhbC1, PhaZ1 and PhaP1. This approach allowed us to determine the main proteins involved in synthesis and degradation as well as the main phasin coating PHB granule. *ΔphbC1* is impaired in producing PHB while *ΔphaP1* deletion mutant showed another protein with low similarity to phasin also associated with PHB. Besides those, novel proteins present in high amount associated with the PHB granule such as a histone-like protein HC2, aconitase AcnA, and a hypothetical protein containing the CYTH-CHAD organo-phosphate recognizing enzyme domains, are likely to play important roles in the metabolism of intracellular PHB granules. The function of these new players will be the object of future work to determine possible links in the PHB metabolism of *H. seropedicae*.

## Supporting Information

Figure S1 **Proteins were identified by peptide mass fingerprinting (PMF) in a MALDI ToF/ToF Autoflex II spectrometer (Bruker Daltonics, USA using positive reflector mode, voltage aceleration of 20 kV, intervals of ion extraction of 150 ns and aquisition of 800–3200 m/z.** Spectra analyses were performed using the FlexAnalysis 3.0 (Bruker Daltonics). Data on peptide fragmentation (MS/MS) was obtained using the LIFT method of FlexControl 3.0 (Bruker Daltonics). Database search was carried out using Mascot 2.3 against a local database of *Herbaspirillum seropedicae* SmR1 strain predicted proteins. Error tolerance for PMF was 100 ppm and 0.3 Da.
(DOC)

Figure S2 **Prediction of secondary structure for the** ***H. seropedicae*** **phasin proteins was obtained using the PSIPRED Protein Sequence Analysis Workbench at (** http://bioinf.cs.ucl.ac.uk/psipred/ **).**
(DOC)
