# Supplementary material for: Identification of Proteins Associated with Polyhydroxybutyrate Granules from Herbaspirillum seropedicae SmR1 - Old Partners, New Players
Source: PLoS One. 2013 Sep 25;8(9):e75066. doi: 10.1371/journal.pone.0075066 (PMC3783465; doi:10.1371/journal.pone.0075066)

Aconitase hydratase (AcnA)

| Match to: **Hsero_2979** | |  |  |  | Score: **70** | |
| --- | --- | --- | --- | --- | --- | --- |
| Id.: acnA aconitate hydratase protein | | |  |  | UniProt: D8IZX7 | |
| Number of mass values searched: **5** | | |  |  |  |  |
| Number of mass values matched: **5** | | |  |  |  |  |
| Sequence Coverage: **7%** | |  |  |  |  |  |
| **Start - End** | **Observed** | **Mr(expt)** | **Mr(calc)** | **ppm** | **Miss** | **Sequence** |
| 77 - 86 | 1144.7078 | 1143.7005 | 1143.6288 | 63 | 0 | R.VDEIPFVVAR.V |
| 257 - 273 | 1844.0387 | 1843.0314 | 1843.0302 | 1 | 0 | R.EGVTATDLVLTITELLR.Q |
| 281 - 297 | 1933.9259 | 1932.9186 | 1932.9105 | 4 | 0 | K.FVEFFGEGTESLSLTDR.A |
| 847 - 864 | 2018.1017 | 2017.0944 | 2017.0956 | -1 | 0 | K.GLEGEIKPQQEATLVIHR.K |
| 871 - 877 | 8706.328 | 869.6255 | 869.5698 | 64 | 1 | K.EVKLLLR.I |


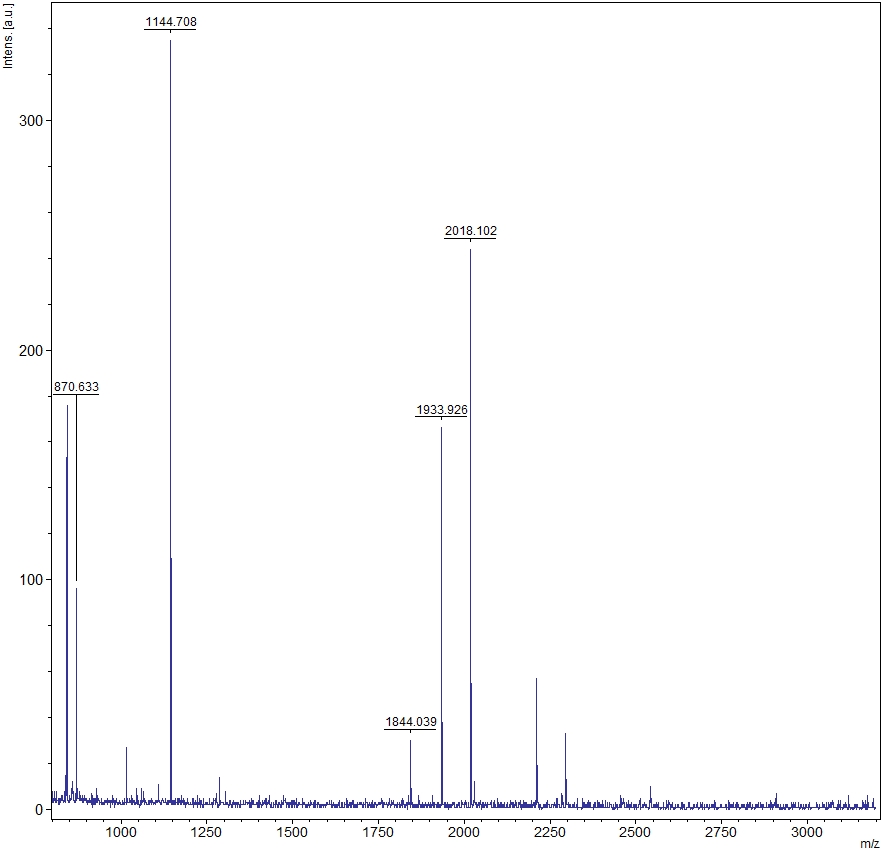


PHB synthase

| Match to: **Hsero_2999** | |  |  |  | Score: **63** | |
| --- | --- | --- | --- | --- | --- | --- |
| Id.: phbC poly(3-hydroxyalkanoate) synthase | | | |  | UniProt: D8J0D1 | |
| Number of mass values searched: **4** | | |  |  |  |  |
| Number of mass values matched: **4** | | |  |  |  |  |
| Sequence Coverage: **9%** | |  |  |  |  |  |
| **Start - End** | **Observed** | **Mr(expt)** | **Mr(calc)** | **ppm** | **Miss** | **Sequence** |
| 186 - 200 | 1632,7903 | 1631,783 | 1631,754 | 18 | 0 | K.GHVSQTDETAFEVGR.D |
| 243 - 256 | 1714,9717 | 1713,9644 | 1713,9202 | 26 | 0 | K.FYILDLQPHNSLVR.Y |
| 257 - 271 | 1835,9823 | 1834,975 | 1834,9366 | 21 | 0 | R.YTVEQGHTVFLISWR.N |
| 456 - 466 | 1222,7201 | 1221,7128 | 1221,6506 | 51 | 0 | K.INAPAFIYASR.D |


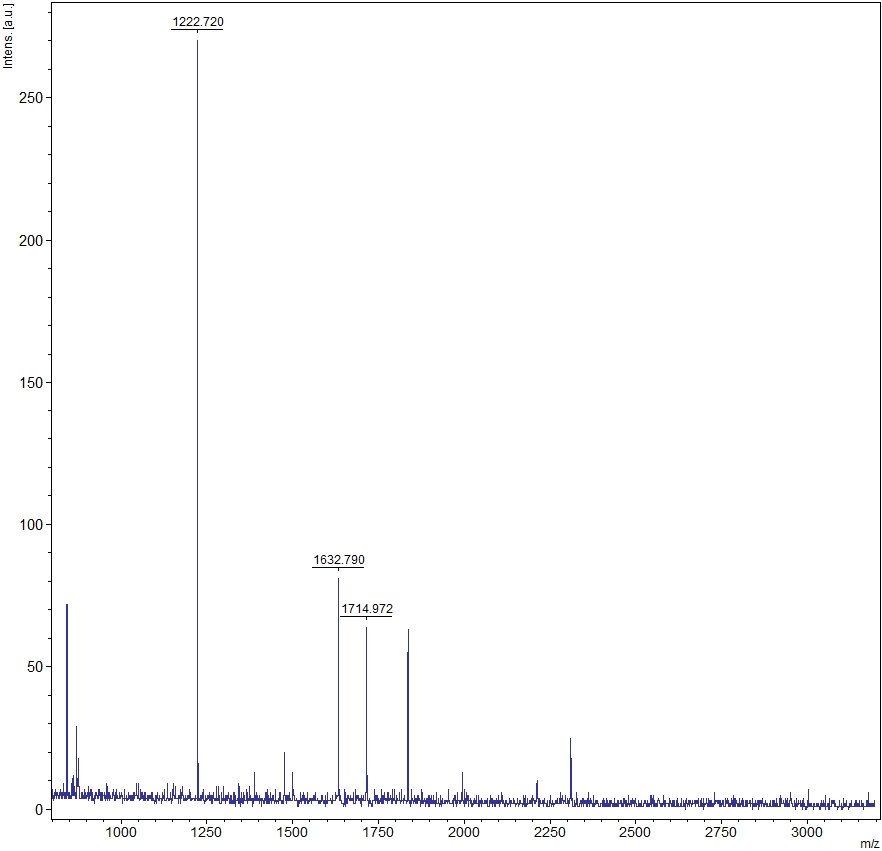


Non-conserved hypothetical protein Hsero_3471

| Match to: **Hsero_3471** | |  |  |  | Score: **62** | |
| --- | --- | --- | --- | --- | --- | --- |
| Id.: Conserved hypothetical protein | | |  |  | UniProt.: D8IPQ3 | |
| Number of mass values searched: **8** | | |  |  |  |  |
| Number of mass values matched: **5** | | |  |  |  |  |
| Sequence Coverage: **11%** | |  |  |  |  |  |
| **Start - End** | **Observed** | **Mr(expt)** | **Mr(calc)** | **ppm** | **Miss** | **Sequence** |
| 77 - 84 | 1043.6868 | 1042.6795 | 1042.5923 | 84 | 1 | R.RLQALWEK.L |
| 89 - 105 | 1891.9657 | 1890.9584 | 1890.9476 | 6 | 0 | K.YGTYAIDGVVAHEELVR.I |
| 189 - 200 | 1314.8518 | 1313.8445 | 1313.7568 | 67 | 0 | R.SLSHHLALLPAR.S |
| 387 - 396 | 1348.6677 | 1347.6604 | 1347.6095 | 38 | 0 | K.WWEDIDISER.H |
| 487 - 500 | 1441.7793 | 1440.7720 | 1440.7184 | 37 | 0 | R.SIGLWGEAGAPMPR.Q |
| **No match to:** 813.5108, 857.0757, 870.6291 | | | |  |  |  |


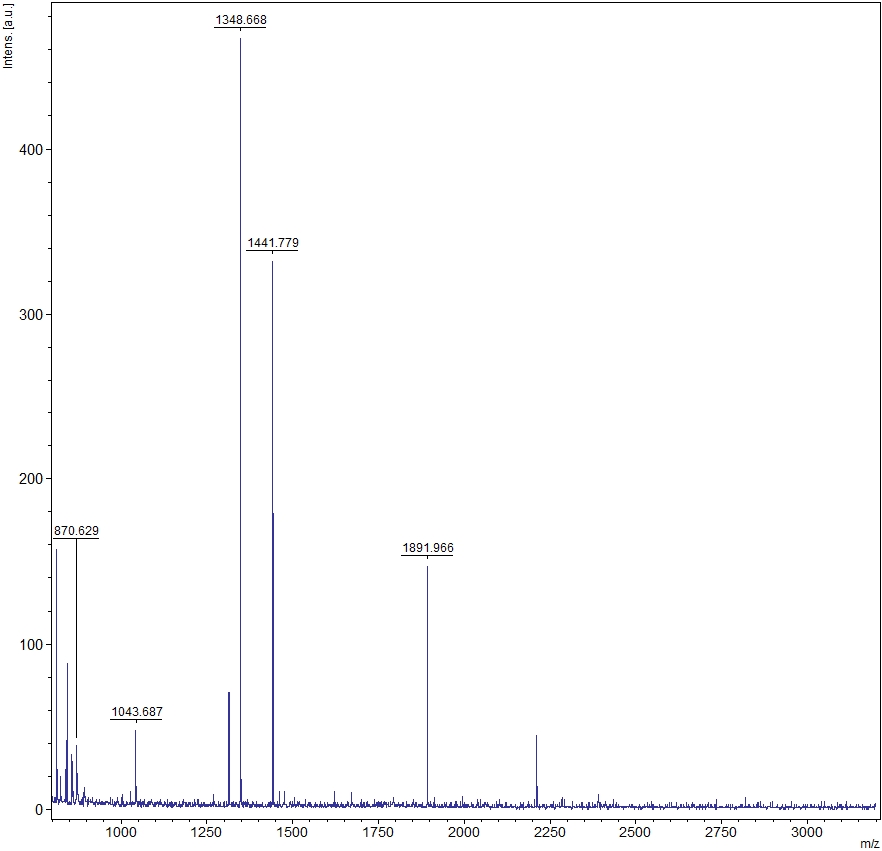


Conserved hypothetical protein Hsero_4241

| Match to: **Hsero_4241** | |  |  |  | Score: **118** | |
| --- | --- | --- | --- | --- | --- | --- |
| Id.: Conserved hypothetical protein | | |  |  | UniProt.: D8IUH8 | |
| Number of mass values searched: **12** | | |  |  |  |  |
| Number of mass values matched: **9** | | |  |  |  |  |
| Sequence Coverage: **20%** | |  |  |  |  |  |
| **Start - End** | **Observed** | **Mr(expt)** | **Mr(calc)** | **ppm** | **Miss** | **Sequence** |
| 7 - 19 | 1450.9231 | 1449.9158 | 1449.8456 | 48 | 0 | K.LLIAPAHTQAFIR.H |
| 70 - 81 | 1095.6539 | 1094.6467 | 1094.5581 | 81 | 0 | K.AGGGVSGGLHQR.H |
| 82 - 99 | 2107.0835 | 2106.0762 | 2106.0382 | 18 | 0 | R.HEWESLVEGEQPDLAVLR.E |
| 100 - 110 | 1258.6772 | 1257.6700 | 1257.5990 | 56 | 0 | R.ELVDSDAPWAR.K |
| 171 - 183 | 1321.7689 | 1320.7616 | 1320.7038 | 44 | 0 | K.SGEATSLLGFALR.L |
| 201 - 209 | 1092.6593 | 1091.6520 | 1091.5512 | 92 | 0 | R.GYTLFHQAR.Q |
| 343 - 355 | 1357.8464 | 1356.8392 | 1356.7262 | 83 | 0 | K.EHALAVHAVASPR.Y |
| 356 - 365 | 1252.7717 | 1251.7645 | 1251.6976 | 53 | 0 | R.YTVLVLQFNR.W |
| 484 - 492 | 1027.6552 | 1026.6479 | 1026.5611 | 85 | 0 | R.QAPSIAWVR.G |
| **No match to:** 870.6499, 1043.6385, 1475.8210 | | | |  |  |  |


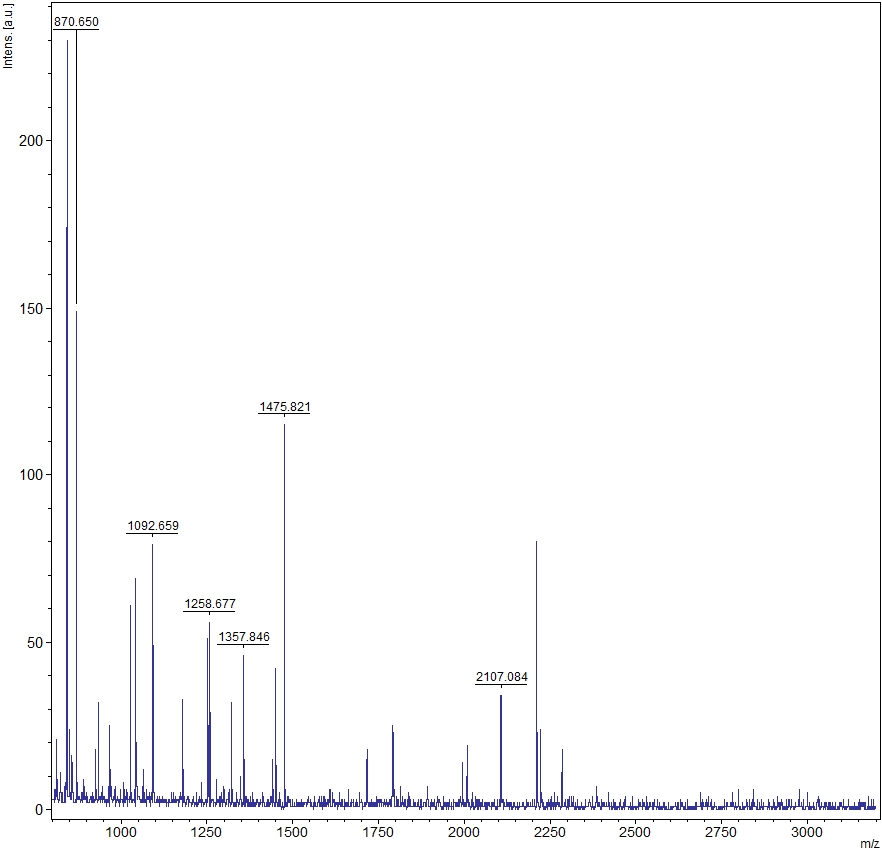


Leucyl aminopeptidase

| Match to: **Hsero_3109** | |  |  |  | Score: **86** | |
| --- | --- | --- | --- | --- | --- | --- |
| Id.: pepA leucyl aminopeptidase protein | | |  |  | UniProt.: D8J0N7 | |
| Number of mass values searched: **7** | | |  |  |  |  |
| Number of mass values matched: **6** | | |  |  |  |  |
| Sequence Coverage: **14%** | |  |  |  |  |  |
| **Start - End** | **Observed** | **Mr(expt)** | **Mr(calc)** | **ppm** | **Miss** | **Sequence** |
| 19 - 30 | 1291.7838 | 1290.7765 | 1290.6932 | 65 | 0 | K.TGALVVGIYENR.K |
| 51 - 65 | 1500.8977 | 1499.8904 | 1499.8308 | 40 | 0 | K.SGDISGKPGSTLLLR.G |
| 90 - 100 | 1109.7056 | 1108.6983 | 1108.5877 | 100 | 0 | K.SFATASSALVR.T |
| 123 - 129 | 904.5660 | 903.5587 | 903.4814 | 86 | 0 | R.DLTWTIR.N |
| 214 - 224 | 1232.7360 | 1231.7287 | 1231.6561 | 59 | 0 | K.QFGLGVEVLDR.K |
| 397 - 413 | 1736.9072 | 1735.9000 | 1735.8489 | 29 | 0 | R.EDEAHDALANALLAAGR.D |
| **No match to:** 1045.6648 | |  |  |  |  |  |


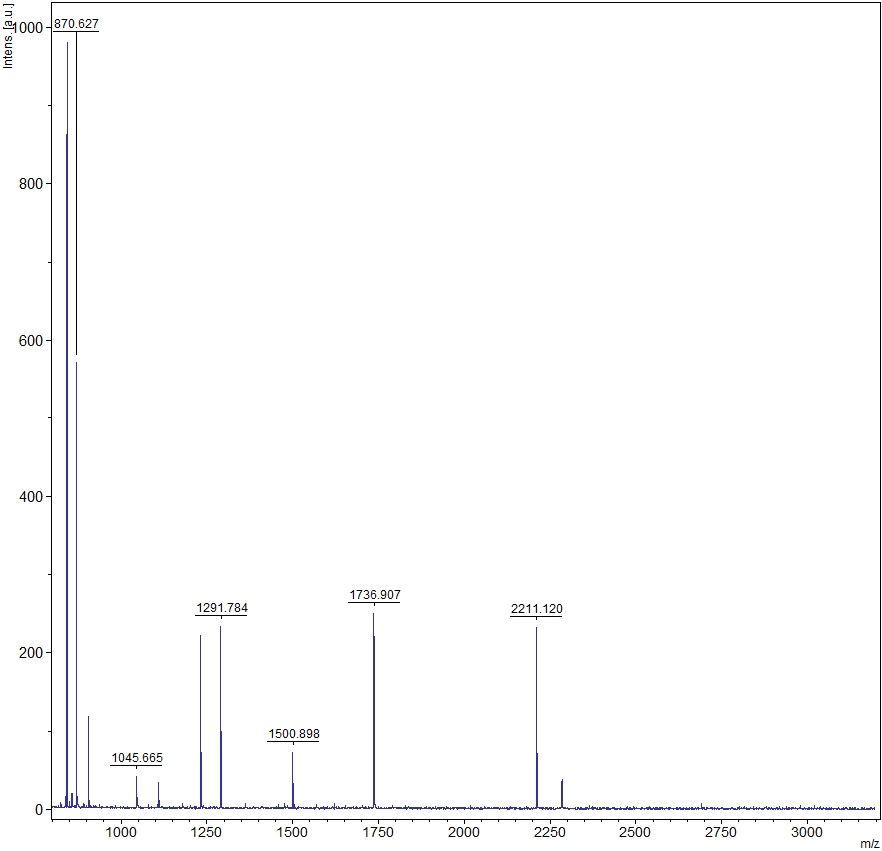


PHB depolymerase (PhaZ1)

| Match to: **Hsero_1622** | |  |  |  | Score: **206** | |
| --- | --- | --- | --- | --- | --- | --- |
| **Id.: PhaZ depolymerase** | |  |  |  | UniProt.: D8IQM4 | |
| Number of mass values searched: **13** | | |  |  |  |  |
| Number of mass values matched: **12** | | |  |  |  |  |
| Sequence Coverage: **39%** | |  |  |  |  |  |
| **Start - End** | **Observed** | **Mr(expt)** | **Mr(calc)** | **ppm** | **Miss** | **Sequence** |
| 1 - 10 | 1316.7495 | 1315.7422 | 1315.6707 | 54 | 0 | -.MLYQLHELNR.A |
| 26 - 42 | 1899.0989 | 1898.0916 | 1897.9687 | 65 | 0 | K.LFSDPVSPLAHTPFSQR.I |
| 43 - 52 | 1168.6887 | 1167.6814 | 1167.6288 | 45 | 0 | R.IAAGYELLYR.L |
| 106 - 124 | 2025.3086 | 2024.3013 | 2024.1531 | 73 | 0 | K.QPTVLVVAPLSGHHSTLLR.E |
| 129 - 145 | 2046.1815 | 2045.1742 | 2044.9854 | 92 | 0 | R.ALLQEHDVYITDWTDAR.M |
| 204 - 215 | 1266.6268 | 1265.6196 | 1265.5566 | 50 | 0 | K.SMTMMGGPIDAR.K |
| 252 - 268 | 1904.1128 | 1903.1055 | 1902.9563 | 78 | 0 | K.VYPGFLQHAGFVAMNPR.R |
| 270 - 282 | 1727.8643 | 1726.8570 | 1726.7787 | 45 | 0 | R.HAQSHWDFYMHLR.D |
| 283 - 294 | 1329.6353 | 1328.6280 | 1328.5593 | 52 | 1 | R.DGDDASAEEHRK.F |
| 316 - 325 | 1208.6992 | 1207.6919 | 1207.6350 | 47 | 0 | K.VVFQDFNLAR.G |
| 326 - 341 | 1869.0809 | 1868.0737 | 1867.9792 | 51 | 0 | R.GTWEIEGQLVRPQDIK.S |
| 384 - 393 | 1064.5764 | 1063.5691 | 1063.5199 | 46 | 0 | K.AGHYGIFSGR.R |
| **No match to:** 1291.7506 | |  |  |  |  |  |


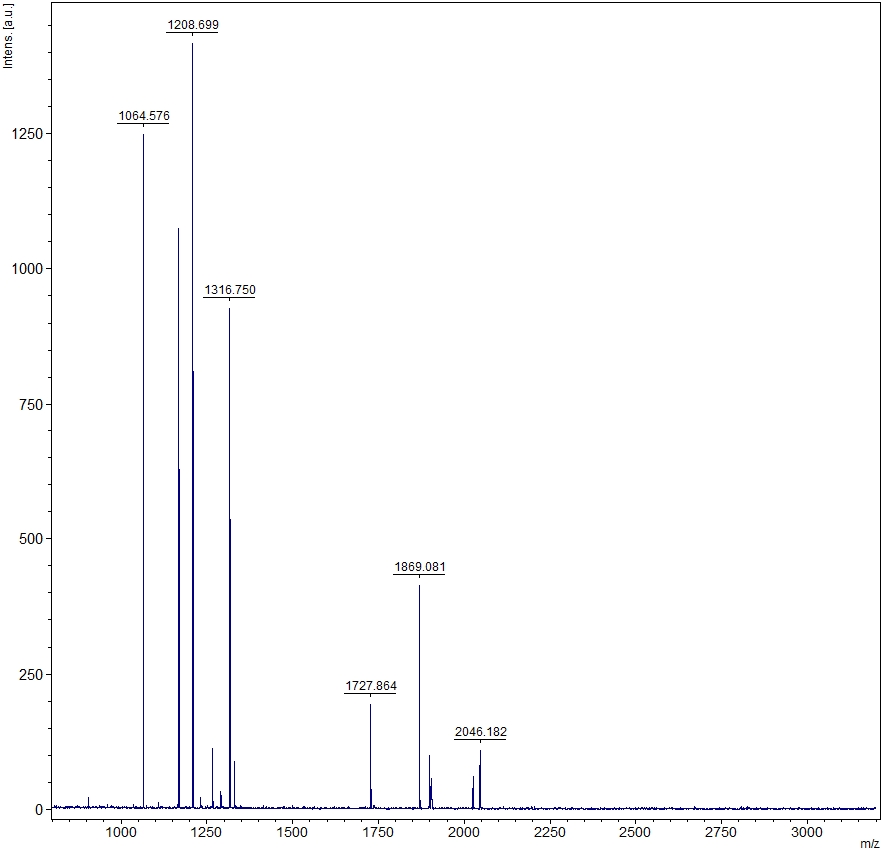


Outer membrane porin Hsero_4295

| Match to: **Hsero_4295** | |  |  |  | Score: **120** | |
| --- | --- | --- | --- | --- | --- | --- |
| Id.: outer membrane (porin) protein | | |  |  | UniProt.: D8IUN2 | |
| Number of mass values searched: **11** | | |  |  |  |  |
| Number of mass values matched: **8** | | |  |  |  |  |
| Sequence Coverage: **33%** | |  |  |  |  |  |
| **Start - End** | **Observed** | **Mr(expt)** | **Mr(calc)** | **ppm** | **Miss** | **Sequence** |
| 55 - 66 | 1234.7612 | 1233.7540 | 1233.6466 | 87 | 0 | K.FGINSGVIQGSR.I |
| 112 - 127 | 1518.9689 | 1517.9616 | 1517.8566 | 69 | 0 | K.SVVGLSGGFGTVLLGR.Q |
| 204 - 219 | 1801.9501 | 1800.9428 | 1800.8682 | 41 | 0 | K.YDNGPLGLGINYYQSK.A |
| 252 - 265 | 1508.8492 | 1507.8420 | 1507.7783 | 42 | 0 | K.SLNVVASYQFGPAR.V |
| 294 - 310 | 1815.9891 | 1814.9819 | 1814.8938 | 49 | 0 | K.ADTYEIGTAYSLSPSLK.L |
| 331 - 344 | 1555.9596 | 1554.9523 | 1554.8293 | 79 | 0 | K.LTQISLGADYFLSK.R |
| 346 - 356 | 1316.7466 | 1315.7393 | 1315.6231 | 88 | 0 | R.TDLYAFVSNMR.A |
| 357 - 384 | 2658.3889 | 2657.3816 | 2657.3257 | 21 | 0 | R.ASDIQNPGVTGGATGSDASQTAIITGIR.H |
| **No match to:** 870.7104, 872.6033, 981.7235 | | |  |  |  |  |


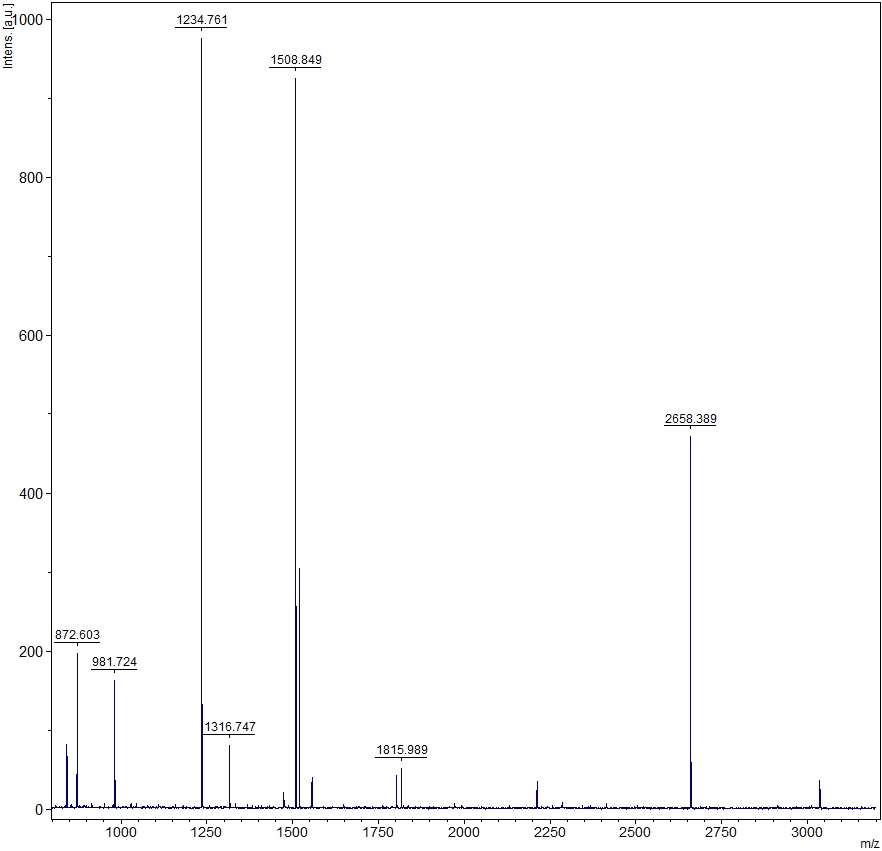


HC2 histone-like protein

| Match to: **Hsero_0382** | |  |  |  | Score: **77** | |
| --- | --- | --- | --- | --- | --- | --- |
| Id.: hypothetical protein | |  |  |  | UniProt.: D8IWA9 | |
| Number of mass values searched: * | | |  |  |  |  |
| Number of mass values matched: ***** | | |  |  |  |  |
| Sequence Coverage: **6%** | |  |  |  |  |  |
| **Start - End** | **Observed** | **Mr(expt)** | **Mr(calc)** | **ppm** | **Miss** | **Sequence** |
| **265 - 282** | **1922.9400** | 1921.9327 | 1921.9799 | -25 | 0 | K.TVLNPAAAWPFPTGNNRP.- (Ions score 77) |
| *** - protein identified only by MS/MS analysis** | | | |  |  |  |


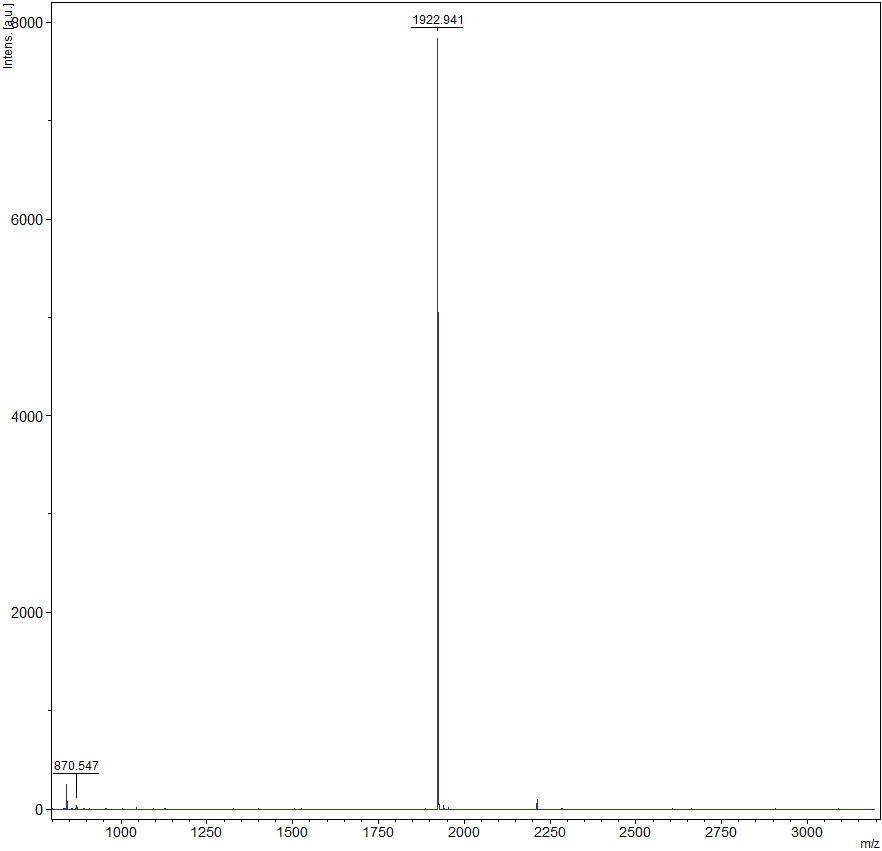


HC2 histone-like protein (Hsero_0382): MS/MS


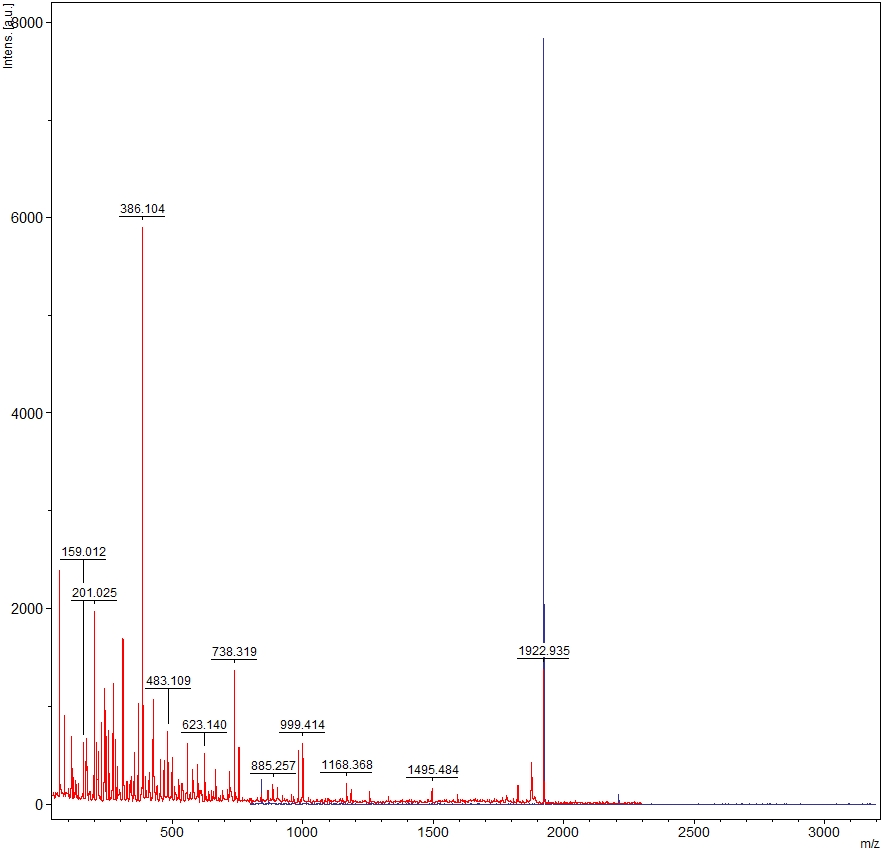


Outer membrane porin (OmpA) Hsero_3696

| Match to: **Hsero_3696** | |  |  |  | Score: **60** | |
| --- | --- | --- | --- | --- | --- | --- |
| Number of mass values searched: **3** | | |  |  | UniProt.:D8IR48 |  |
| Number of mass values matched: **3** | | |  |  |  |  |
| Sequence Coverage: **17%** | |  |  |  |  |  |
| **Start - End** | **Observed** | **Mr(expt)** | **Mr(calc)** | **ppm** | **Miss** | **Sequence** |
| 30 - 41 | 1417.7852 | 1416.7779 | 1416.7473 | 22 | 0 | K.VRPNSLYLQDGR.D |
| 90 - 102 | 1509.7186 | 1508.7114 | 1508.6824 | 19 | 0 | K.VTYAADAFFDFDK.A |
| 193 - 202 | 1157.7009 | 1156.6937 | 11566564 | 32 | 1 | R.RVEIEVVGTR.K |


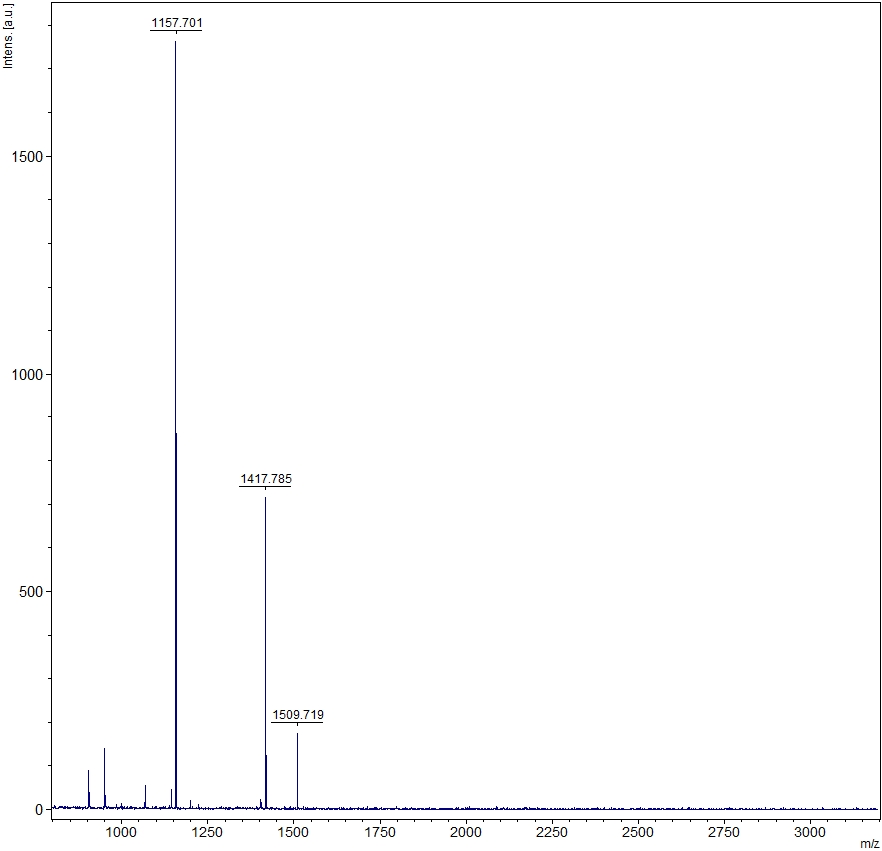


Phasin (PhaP1)

| Match to: **Hsero_1639** | |  |  |  | Score: **163** | |
| --- | --- | --- | --- | --- | --- | --- |
| Id.: phasin family protein protein | |  |  |  | UniProt.: D8IQP1 | |
| Number of mass values searched: **26** | | |  |  |  |  |
| Number of mass values matched: **11** | | |  |  |  |  |
| Sequence Coverage: **79%** | |  |  |  |  |  |
| **Start - End** | **Observed** | **Mr(expt)** | **Mr(calc)** | **ppm** | **Miss** | **Sequence** |
| 2 - 13 | 1317.6180 | 1316.6108 | 1316.6248 | -11 | 0 | M.TTYTEQFSAAAK.A |
| 14 - 29 | 1661.9294 | 1660.9222 | 1660.8784 | 26 | 0 | K.ANAEAQIALFSQLASK.T |
| 47 - 58 | 1262.6105 | 1261.6032 | 1261.6150 | -9 | 0 | R.STLEESQAAAQK.L |
| 64 - 82 | 2130.2192 | 2129.2120 | 2129.0429 | 79 | 0 | K.DPQEFFALTSAHAQPTLEK.S |
| 89 - 103 | 1618.8319 | 1617.8246 | 1617.8362 | -7 | 0 | R.HLSGIFSSTQAELTK.A |
| 104 - 114 | 1171.6088 | 1170.6015 | 1170.5993 | 2 | 0 | K.AAEAQIAEVNR.K |
| 104 - 115 | 1299.6866 | 1298.6794 | 1298.6942 | -11 | 1 | K.AAEAQIAEVNRK.V |
| 116 - 125 | 1074.6191 | 1073.6119 | 1073.5791 | 31 | 0 | K.VVAMIDEVAK.N |
| 126 - 139 | 1370.7156 | 1369.7083 | 1369.7201 | -9 | 0 | K.NAPAGSEQAVSVLK.S |
| 140 - 154 | 1603.6929 | 1602.6856 | 1602.7348 | -31 | 0 | K.SAIGNMSAGYEQFTK.N |
| 158 - 180 | 2359.3701 | 2358.3628 | 2358.1485 | 91 | 0 | K.QAAEVLEANVTNAVEQMSQAGAK.V |
| **No match to:** 1196.6084, 1227.6200, 1242.6803, 1444.7607, 1588.7870, 1631.5554, 1640.8170, 1644.9208, | | | | | | |
| 1683.9587, 2036.1306, 2116.1526, 2152.2104, 2342.1660, 2345.6343, 2375.4460 | | | | | | |


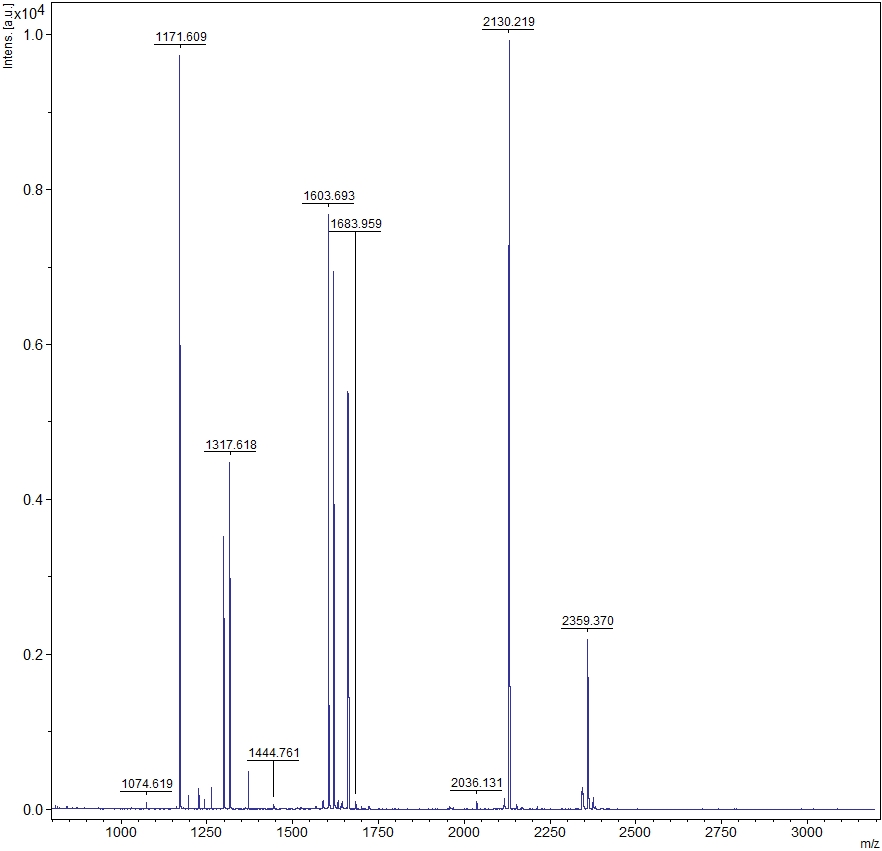


PhbF (PHB regulatory protein)

| Match to: **Hsero_2997** | |  |  |  | Score: **79** | |
| --- | --- | --- | --- | --- | --- | --- |
| Id.: (PhbF) protein | |  |  |  | UniProt.: D8J0C9 | |
| Number of mass values searched: **6** | | |  |  |  |  |
| Number of mass values matched: **6** | | |  |  |  |  |
| Sequence Coverage: **48%** | |  |  |  |  |  |
| **Start - End** | **Observed** | **Mr(expt)** | **Mr(calc)** | **ppm DELTA** | **Miss** | **Sequence** |
| 20 - 35 | 1847.9302 | 1846.9229 | 1846.9200 | 0.0029 | 0 | R.LYDTQTSSYITLTDVK.Q |
| 36 - 50 | 1719.8839 | 1718.8766 | 1718.8727 | 0.0040 | 0 | K.QLVLDNEEFTVVDAK.S |
| 86 - 101 | 1892.8441 | 1891.8368 | 1891.8055 | 0.0313 | 0 | R.YYGHAMQGMMGNYLEK.N |
| 102 - 112 | 1333.7244 | 1332.7171 | 1332.7038 | 0.0133 | 0 | K.NIQTFIDIQNK.L |
| 155 - 169 | 1853.8918 | 1852.8846 | 1852.8811 | 0.0034 | 0 | K.NLFIQMQEQMQSQTK.N |
| 170 - 187 | 1881.8748 | 1880.8675 | 1880.8516 | 0.0159 | 0 | K.NMFGTFPFGNPGAGPDQK.K |


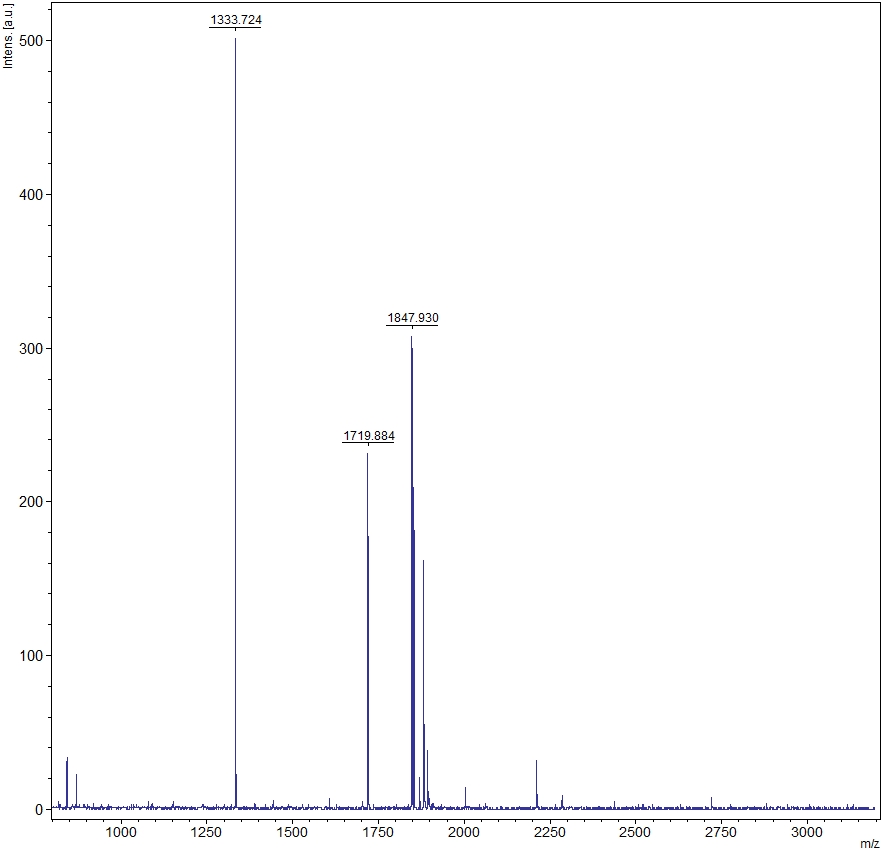


PhaP2 (phasin 2) - Hsero_4759

| Match to: **Hsero_4759 (PhaP2)** | |  |  |  | Score: **130** | |
| --- | --- | --- | --- | --- | --- | --- |
| Number of mass values searched: **14** | | |  |  | Uniprot: D8IZ16 | |
| Number of mass values matched: **8** | | |  |  |  |  |
| Sequence Coverage: **60%** | |  |  |  |  |  |
| **Start - End** | **Observed** | **Mr(expt)** | **Mr(calc)** | **ppm** | **Miss** | **Sequence** |
| 1 - 13 | 1523,7509 | 1522,7436 | 1522,6762 | 44 | 0 | -.MFSYQDQFSAATK.A Oxidation (M) |
| 14 - 29 | 1722,0848 | 1721,0776 | 1720,9471 | 76 | 0 | K.ANLQAHLDLINSLTAK.A |
| 64 - 82 | 1967,1448 | 1966,1375 | 1966,0007 | 70 | 0 | K.DPQELLSLATAQAQPGAEK.A |
| 89 - 103 | 1588,958 | 1587,9507 | 1587,8257 | 79 | 0 | R.HLAGIVSSTQAEFTK.A |
| 104 - 114 | 1146,6166 | 1145,6093 | 1145,5676 | 36 | 0 | K.AAEAQIAETSR.K |
| 115 - 125 | 1230,7562 | 1229,7489 | 1229,7231 | 21 | 0 | R.KLSALIDEITK.N |
| 126 - 139 | 1410,7941 | 1409,7868 | 1409,7514 | 25 | 0 | K.NAPPGSEQAVSILK.A |
| 140 - 154 | 1594,9415 | 1593,9343 | 1593,7998 | 84 | 0 | K.ATLTNANAAYEQLSK.N |


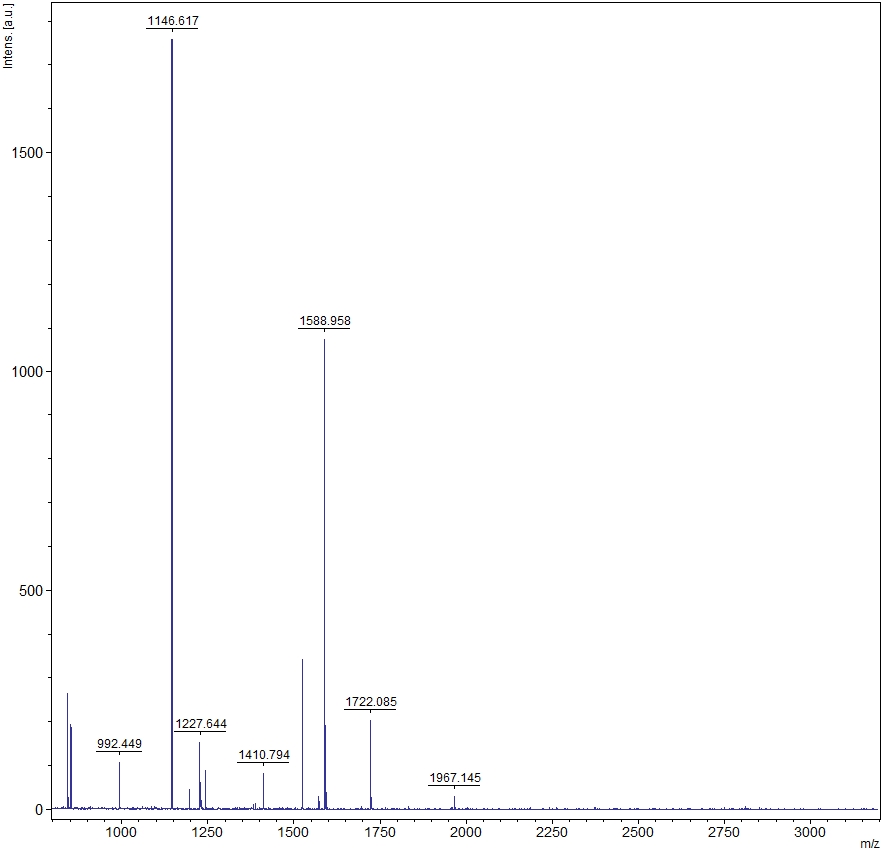


Hypothetical protein Hsero_2402

| Match to: **Hsero_2402** | |  |  |  | Score: **64** | |
| --- | --- | --- | --- | --- | --- | --- |
| Number of mass values searched: **6** | | |  |  | Uniprot: D8IVG2 | |
| Number of mass values matched: **4** | | |  |  |  |  |
| Sequence Coverage: **18%** | |  |  |  |  |  |
| **Start - End** | **Observed** | **Mr(expt)** | **Mr(calc)** | **ppm** | **Miss** | **Sequence** |
| 38 - 47 | 1227,4403 | 1226,433 | 1226,5932 | -131 | 0 | K.VYDLDSNFVR.R |
| 65 - 76 | 1242,5371 | 1241,5298 | 1241,6615 | -106 | 0 | R.TPVEAASLVAER.W |
| 102 - 108 | 853,4692 | 852,462 | 852,4566 | 6 | 0 | K.LNHSVQR.H |
| 109 - 115 | 810,4339 | 809,4266 | 809,4395 | -16 | 0 | R.HAIEVNK.Q |


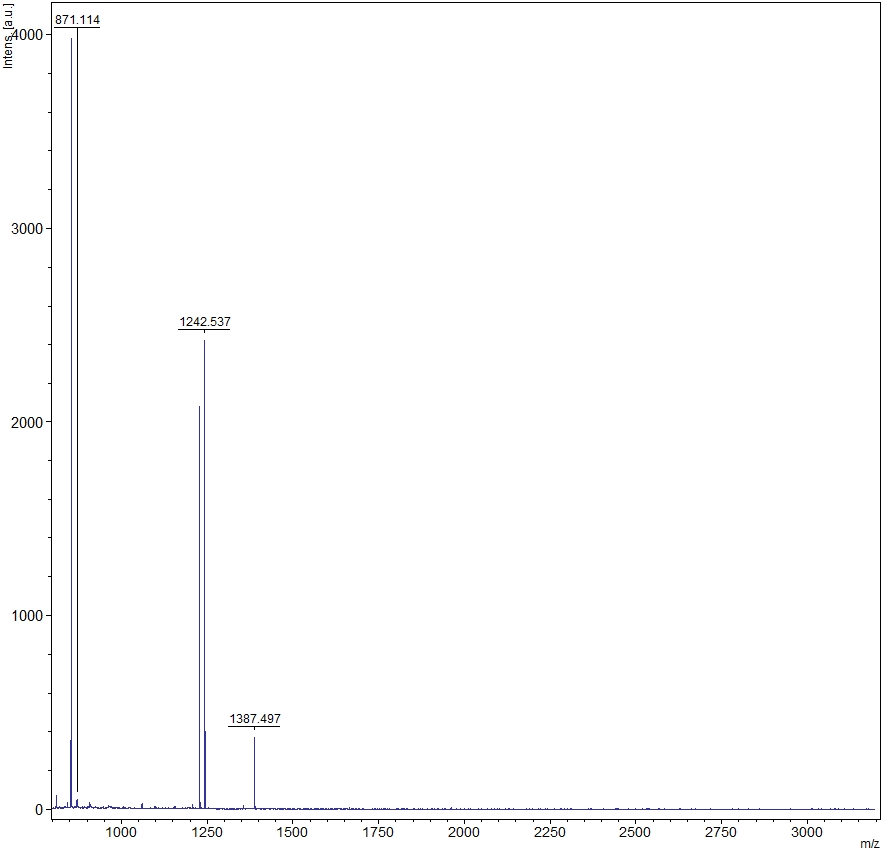

Supplement: Figure S1 — Proteins were identified by peptide mass fingerprinting (PMF) in a MALDI ToF/ToF Autoflex II spectrometer (Bruker Daltonics, USA using positive reflector mode, voltage aceleration of 20 kV, intervals of ion extraction of 150 ns and aquisition of 800–3200 m/z. Spectra analyses were performed using the FlexAnalysis 3.0 (Bruker Daltonics). Data on peptide fragmentation (MS/MS) was obtained using the LIFT method of FlexControl 3.0 (Bruker Daltonics). Database search was carried out using Mascot 2.3 against a local database of Herbaspirillum seropedicae SmR1 strain predicted proteins. Error tolerance for PMF was 100 ppm and 0.3 Da. (DOC) [file pone.0075066.s001.doc]
