# Supplementary figures and images for: Identification of Proteins Associated with Polyhydroxybutyrate Granules from Herbaspirillum seropedicae SmR1 - Old Partners, New Players
Source: PLoS One. 2013 Sep 25;8(9):e75066. doi: 10.1371/journal.pone.0075066 (PMC3783465; doi:10.1371/journal.pone.0075066)

PhaP1 (Hsero_1639)


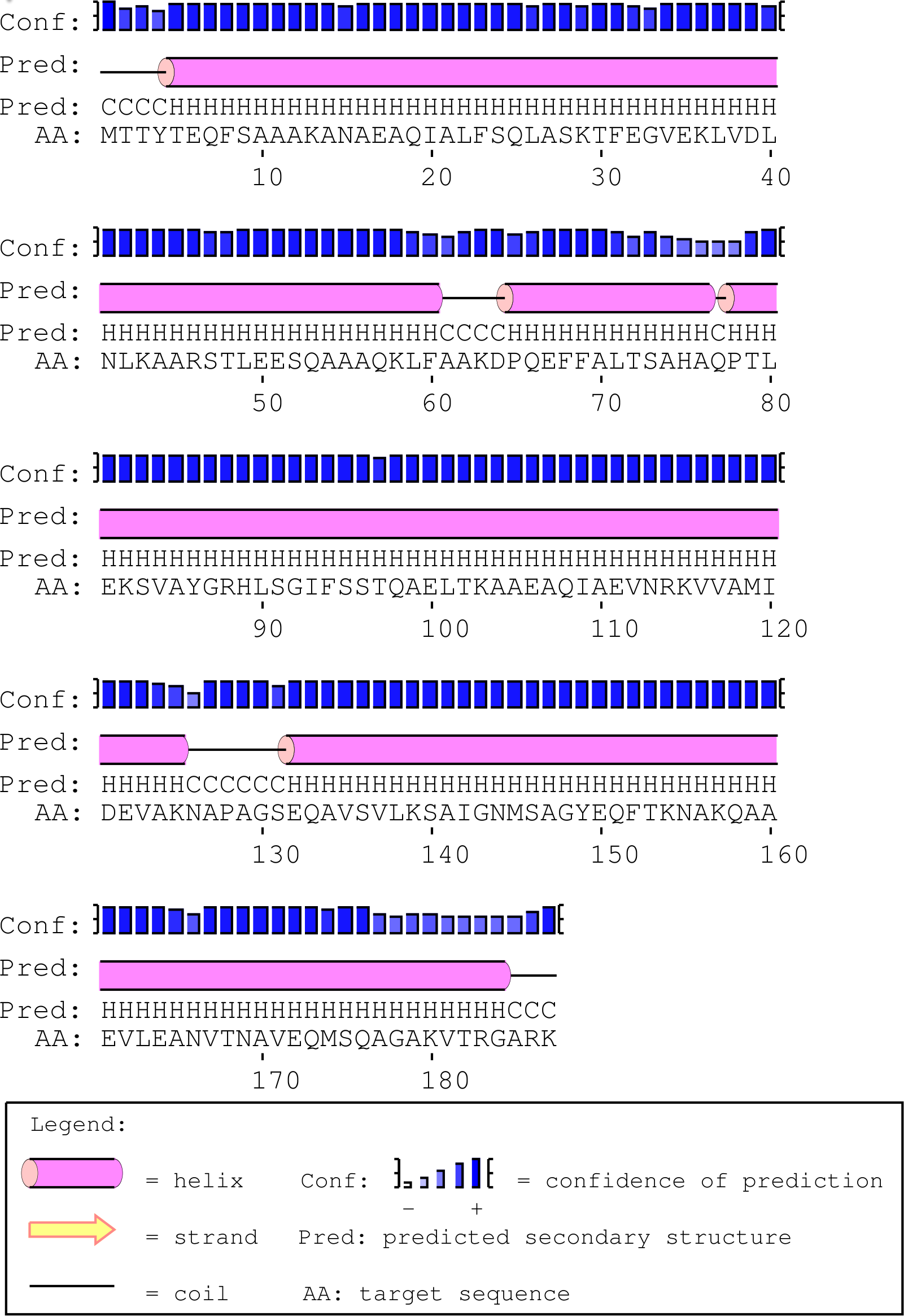


PhaP2 (Hsero_4759)


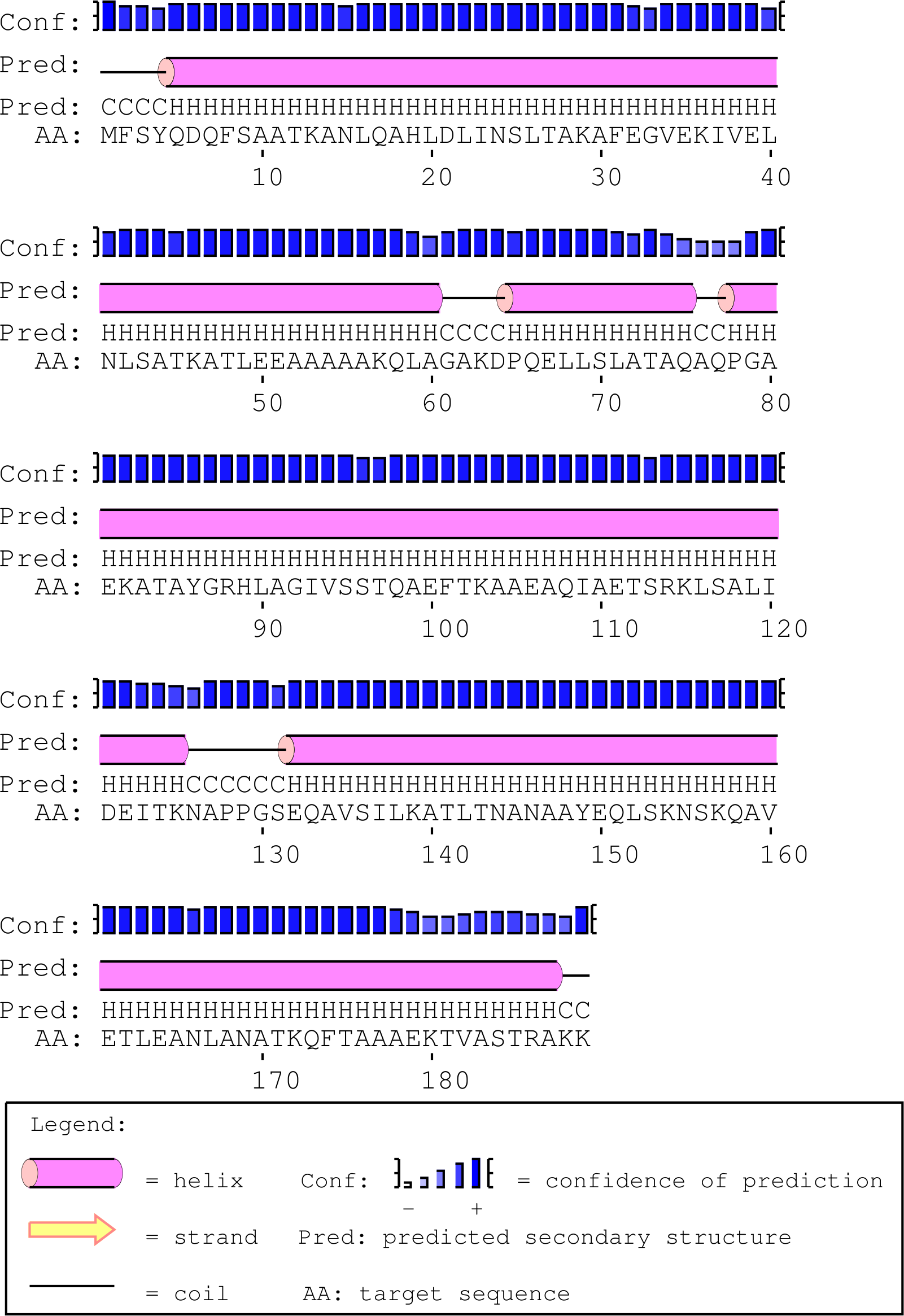


Hsero_2402


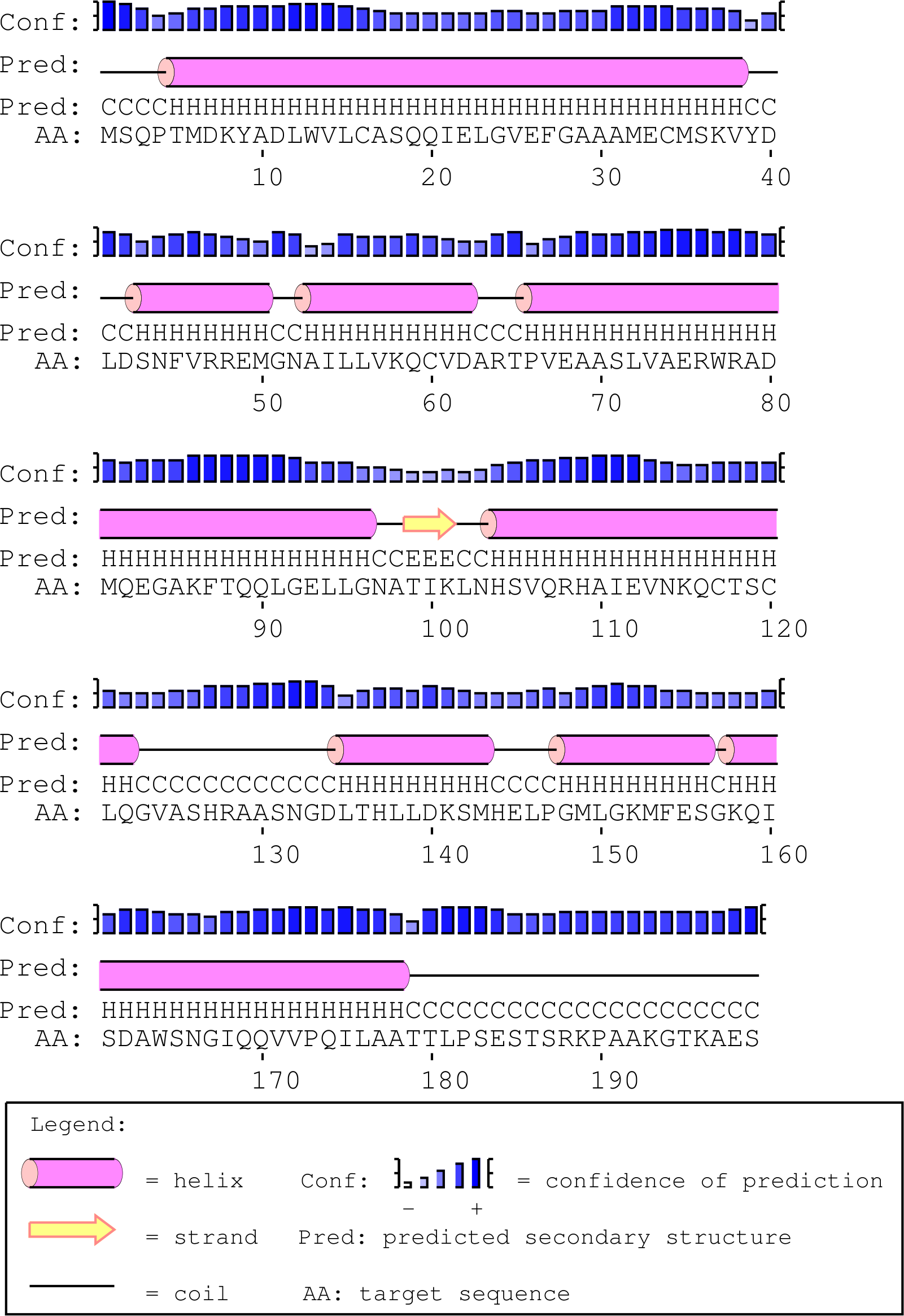

Supplement: Figure S2 — Prediction of secondary structure for the H. seropedicae phasin proteins was obtained using the PSIPRED Protein Sequence Analysis Workbench at ( http://bioinf.cs.ucl.ac.uk/psipred/ ). (DOC) [file pone.0075066.s002.doc]
